# Supplementary material for: Alteration of the Neuromuscular Junction and Modifications of Muscle Metabolism in Response to Neuron-Restricted Expression of the CHMP2Bintron5 Mutant in a Mouse Model of ALS-FTD Syndrome
Source: Biomolecules. 2022 Mar 24;12(4):497. doi: 10.3390/biom12040497 (PMC9025139; doi:10.3390/biom12040497)
Supplement: Supplementary file 1 [file biomolecules-12-00497-s001.zip › biomolecules-1532431-supplementary.pdf]

Supplementary Materials

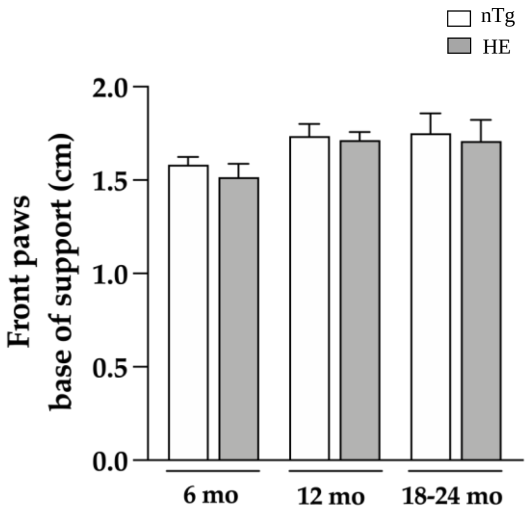

Supplementary Figure S1. The front paws spacing is not altered in HE mice compared to age-matched nTg mice. Gait was analyzed with the Catwalk software as described in the « material and methods » section.
Histograms represent means ± SEM; n = 9–16/group.

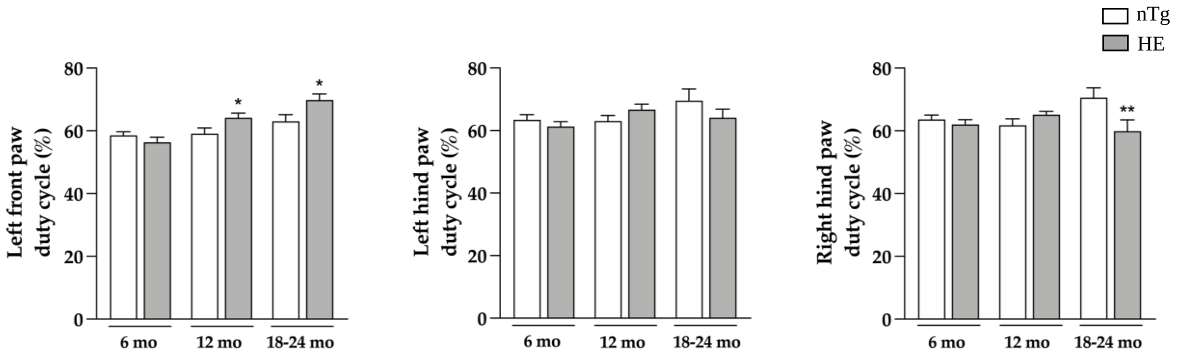

Supplementary Figure S2. The duty cycle of left paws is asymmetrically altered.
The duty cycle of the left front paw (left) increases gradually from 12 months of age in HE mice compared to age-matched nTg mice whereas the duty cycle of the left hind paw (middle) is similar between genotypes. The right hind paw duty cycle (right) decreases at 18–24 months.
Histograms represent means ± SEM; n = 9–16/group. * $p < 0.05$, Student's t test.

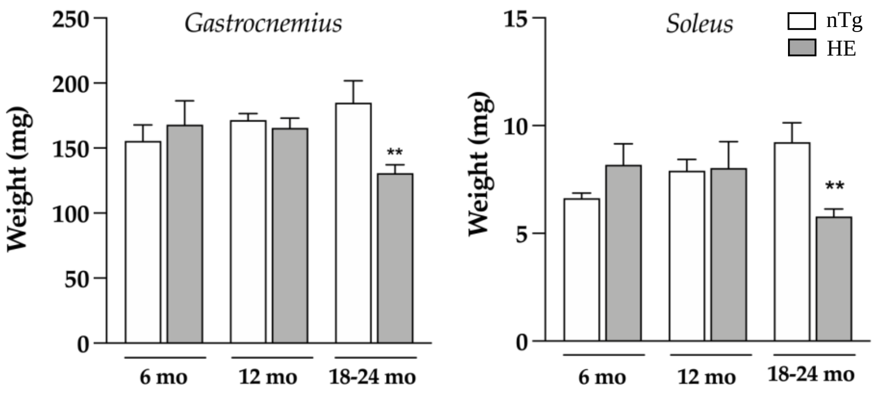

Supplementary Figure S3. Evolution of *gastrocnemius* (left) and *soleus* (right) muscles mass with age. HE mice are compared to age-matched nTg mice.
Histograms represent means ± SEM; n = 4–6/group; ** $p < 0.01$, Student's t test.

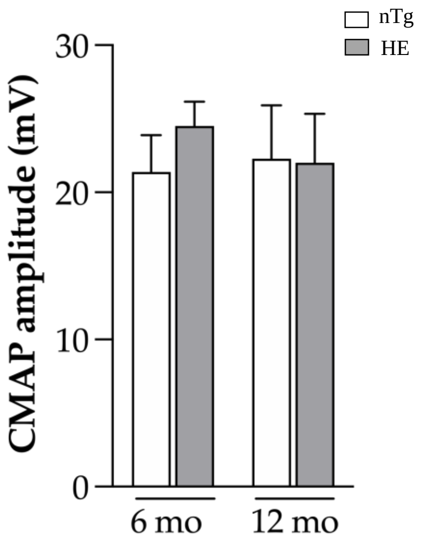

Supplementary Figure S4. CMAP amplitude in nTg and HE mice after a single stimulation of the sciatic nerve at supramaximal intensity at 6 and 12 months. No difference is seen between genotypes.
Histograms represent means ± SEM; n = 4–7/group.

(A) 6 months

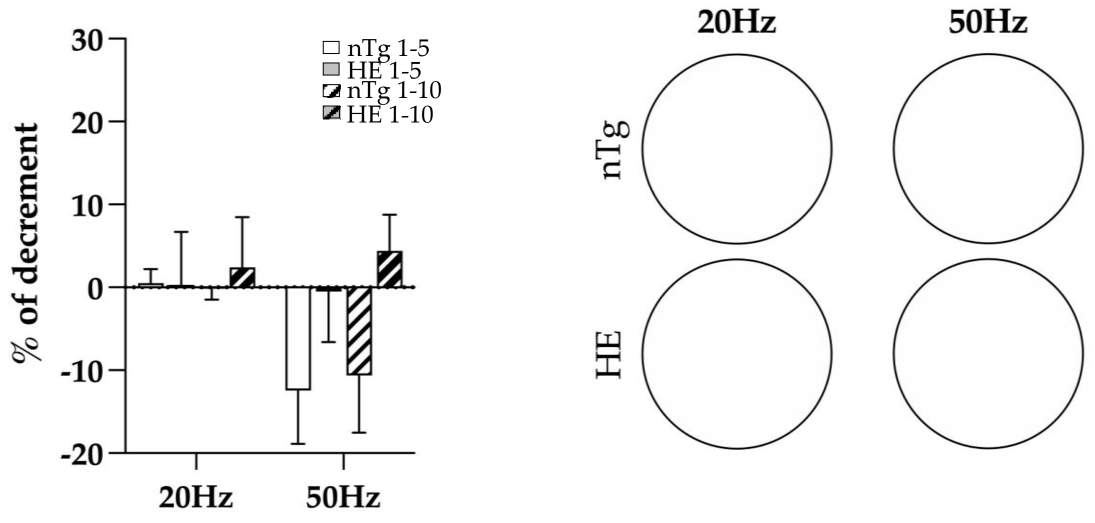

(B) 12 months

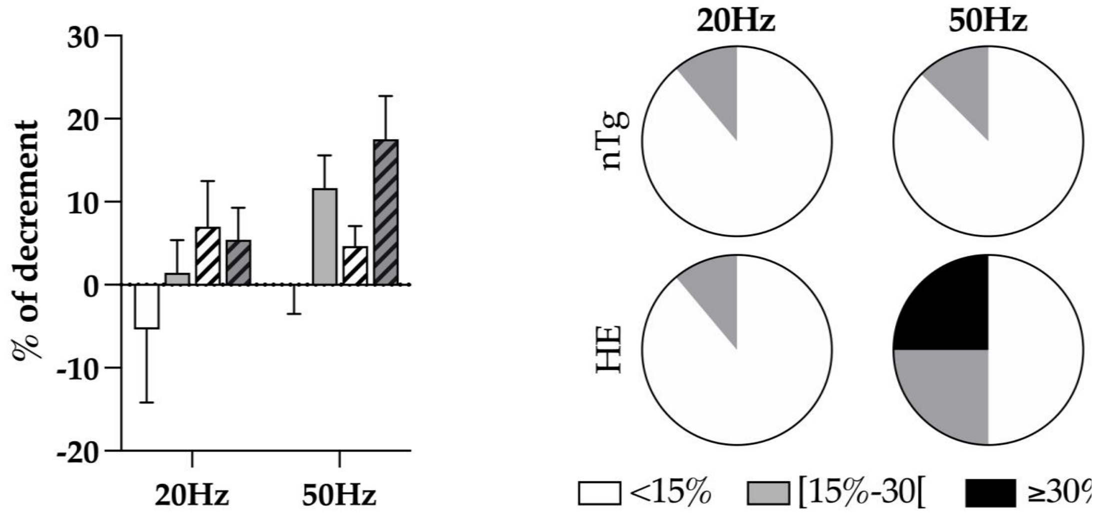

Supplementary Figure S5. CMAP response to repetitive nerve stimulations in mice aged of 6 (A) and 12 (B) months.

Left: Quantification of CMAP decrement between the first and fifth stimulations (plain histograms) or the first and the tenth stimulation (dashed histograms) after electrical stimulations of the sciatic nerve at 20Hz and 50Hz.

Right: Proportion of mice with less than 15% decrement (white), between 15 and 30% decrement (grey) and more than 30% decrement (black) in the nTg and HE mice for each frequency of stimulation.

Histograms represent means ± SEM; n = 4–9/group.

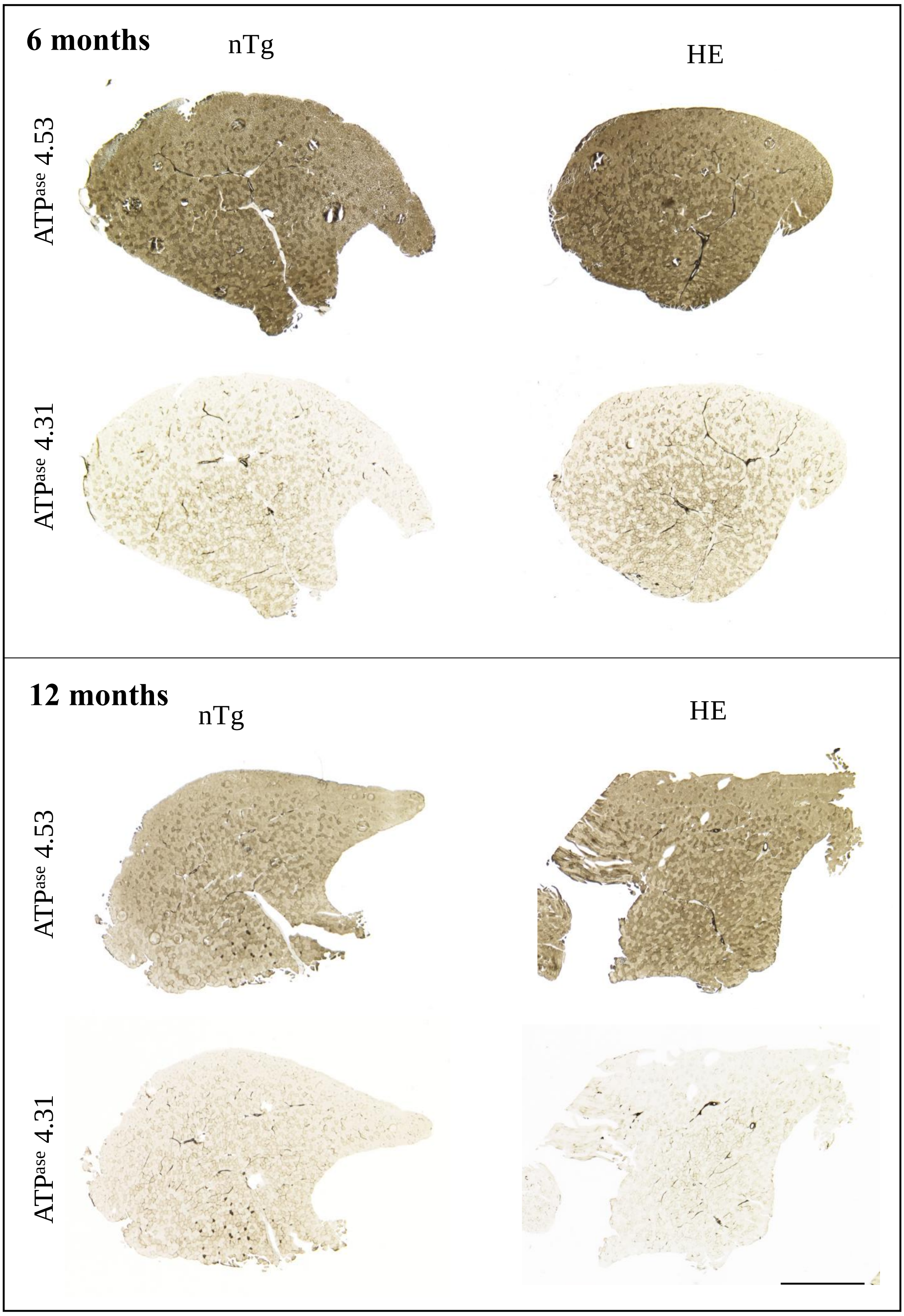

Supplementary Figure S6. Representative micrographs of serial sections of TA after myofibrillar ATPase histochemistry.

Sections were either preincubated at pH 4.53 or at pH 4.31. At pH 4.53 type 1 fibers are the darkest, type 2A fibers are the lightest, type 2B fibers are brown and type 2X fibers appear dark brown. At pH 4.31 type 1 fibers are the darkest, type 2X fibers are intermediate and type 2A and 2B fibers are the lightest. Same magnification is used. Scale bar: 500 μm.

Supplementary Table S1. Sequences of primers used for qPCR studies.

Gene	5′ – 3′ (Forward)	3′ – 5′ (Reverse)
AchR α	CCACAGACTCAGGGGAGAAG	AACGGTGGTGTGTGTTGATG
AchR ε	CAATGCCAATCCAGACACTG	CCCTGCTTCTCCTGACACTC
AchR γ	TGAGTGTGCTCTTGGGGAAA	GACCATACCCATCACCAACCA
Atp5g3	CGCACCCCCGCTCTG	TAGAGCCCTCTCCAGTCCTAGTC
Atrogin-1	AGTGAGGACCGGCTACTGTG	GATCAAACGCTTGCGAATCT
Cox5A	GCTGTCTGTTCCATTCGCTG	AATATGTCACCCAGCGAGCA
H1H2BC	AGTGAGGACCGGCTACTGTG	GATCAAACGCTTGCGAATCT
H2AC	CAACGACGAGGAGCTCAACAAG	GAAGTTTCCGCAGATTCTGTTGC
Lcad	TGGGGACTTGCTCTCAACA	GGCCTGTGCAATTGGAGTA
Musk	GCTGGAAGTGGAGGAAGACA	GATTCAGGAGCACGCAGGAT
MyHC1	GCATCCCTAAAGGCAGGCTC	ACTTCCGGAGGTAAGGAGCA
MyHC2	CGAAGCGAGGCACAAAATGT	TTGCTTGCAAAGGAACTTGGG
MyHC4	ACAGGACAGTGACAAAGAACG	GGTGAAGAGCCGAGAGGTTC
MyHC7	CAAGAGCCGGGACATTGGT	TGTTGCAAAGGCTCCAGGTC
Myoglobin	CAAGCACAAGATCCCGGTCA	TCCAAAGTCCCCGGAATGTC
Parv	ATCAAGAAGGCGATAGGAGCC	GGCCAGAAGCGTCTTTGTT
Pdk4	GCTGGATGTTTGGTGGTTCT	TGCTTTGATTCCTCCCATCC
Pgc1α	TGCTGCTGTTCCTGTTTTC	CCCTGCCATTGTTAAGACC
Pparα	CCAGAGAAGAGGGCCTTGA	CCATCCAGGTATGAGGAGTCTT
Pparδ	ATG-GGG-GAC-CAG-AAC-ACA-C	TCTCAATCAGTGTGCGGTCC
RyR1	GCACACAGTCGTATGTACCTG	CCTCCCCTGTTGCGTCTTC
Sdha	GTGTGAAGTAGGGCAGGTCC	ACAAGGCACTGGCTCGATAC
Tbp	CCAATGACTCCTATGACCCCTA	CAGCCAAGATTCACGGTAGAT
Tfam	GGGAATGTGGAGCGTGCTAA	TGATAGACGAGGGGATGCGA
TnnC1	GGCACAGTGGACTTCGATGA	TTCCCTTTGCTGTCGTCCTT
TnnC2	AAAGAGTTGGGCACCGTGAT	GGCATCCAATTCCTCTTTGG
Uqcrc2	AACTGCTAGAGCCATGAAGC	AAGTTTTAACCTTCGGGGCAAC
